# Mutations in *ampD* cause hyperproduction of AmpC and CmcB β-lactamases and high resistance to β-lactam antibiotics in *Chromobacterium violaceum*

**DOI:** 10.1128/spectrum.00916-25

**Published:** 2025-06-12

**Authors:** Luís Gustavo Laranjeiro, Carlos Eduardo M. Neme, Maristela Previato-Mello, Bianca B. Batista, Isabel Henriques, José F. da Silva Neto

**Affiliations:** 1 Departamento de Biologia Celular e Molecular e Bioagentes Patogênicos, Faculdade de Medicina de Ribeirão Preto, Universidade de São Paulo 28133, Ribeirão Preto, São Paulo, Brazil; 2 Departamento de Ciências da Vida, Centro de Ecologia Funcional, Laboratório Associado TERRA, Faculdade de Ciências e Tecnologia, Universidade de Coimbra 168241 , Coimbra, Coimbra District, Portugal; JMI Laboratories, North Liberty, Iowa, USA

**Keywords:** *Chromobacterium violaceum*, AmpD amidases, β-lactam resistance, β-lactamases, spontaneous mutants

## Abstract

Bacterial resistance to β-lactam antibiotics mediated by β-lactamase enzymes is widespread worldwide. *Chromobacterium violaceum*, an environmental Gram-negative bacterial pathogen, is intrinsically resistant to some β-lactam antibiotics. In this work, we found that mutations in an *ampD* gene, encoding a peptidoglycan-recycling amidase, cause hyperproduction of two chromosomal β-lactamases (AmpC and CmcB), conferring high β-lactam resistance in *C. violaceum*. Susceptibility tests using Δ*ampC*, Δ*cmcB*, and Δ*cmcBΔampC* mutant strains revealed specific susceptibility profiles to penicillin, cephalosporin, and carbapenem β-lactams, suggesting that AmpC is a broad-spectrum β-lactamase (penicillinase and cephalosporinase), while CmcB is a narrow-spectrum metallo-carbapenemase. β-galactosidase assays indicate that the expression of *ampC* and *cmcB* increased in response to β-lactams. We isolated *C. violaceum* spontaneous mutants resistant to the antibiotic ceftazidime and found that most mutants were also resistant to several other β-lactams and overexpressed *ampC* and *cmcB*. DNA sequencing of the three paralog genes encoding the *C. violaceum* AmpD amidases revealed mutations of different types in AmpD1 (CV_0566) in most of the spontaneous mutants, but no mutation was found in AmpD2 or AmpD3. Analysis of single and combined null amidase mutants revealed overexpression of both β-lactamases and increased resistance to β-lactams only in mutants with deleted *ampD1*. When introduced into *ampD1* null or spontaneous mutants, the *ampD1* gene rescued the antibiotic-related phenotypes. The AmpD1 amidase from *C. violaceum* has a unique architecture with an N-terminal acetyltransferase domain. Our work offers new insights into the mechanisms of β-lactamase-mediated antibiotic resistance and opens perspectives to improve the treatment of *C. violaceum* infections.

**IMPORTANCE:**

Resistance to β-lactam antibiotics reduces the options for treating bacterial infections, posing a threat to public health. In this work, we demonstrated that the intrinsic resistance to β-lactam antibiotics in the environmental pathogen *Chromobacterium violaceum* is mediated by two chromosomally encoded β-lactamases, AmpC and CmcB, and revealed the mechanism that contributes to their simultaneous expression. Our data indicate that mutations in the peptidoglycan recycling amidase *ampD1*, but not in its paralogs *ampD2* and *ampD3*, lead to stable overexpression of both β-lactamases and increased resistance to β-lactam antibiotics. Remarkably, AmpD1 possesses a unique N-terminal acetyltransferase domain, suggesting a distinct functional mechanism for this enzyme. Our work offers an explanation for the limited effectiveness of many β-lactams in treating *C. violaceum* infections. Understanding the mechanism of antimicrobial resistance is crucial for developing effective treatments and mitigating the spread of β-lactam-resistant bacteria.

## INTRODUCTION

Antimicrobial resistance has emerged and spread among bacterial pathogens, posing a threat to public health worldwide (1, 2). Resistance to β-lactams, an important class of bactericidal antibiotics that block cell wall synthesis, is typically mediated by β-lactamases in Gram-negative bacteria. These enzymes hydrolyze β-lactams such as penicillins, cephalosporins, monobactams, and carbapenems with variable efficiency, using a conserved serine residue (classes A, C, and D β-lactamases) or zinc as a cofactor (class B metallo-β-lactamases) (3–5). Chromosomally encoded β-lactamases such as AmpC (a class C cephalosporinase) (6) and CphA (a class B, subclass B2 narrow-spectrum carbapenemase) (7–9) are important determinants of β-lactam resistance in many bacterial pathogens, including *Pseudomonas aeruginosa*, *Stenotrophomonas maltophilia*, *Aeromonas* spp., and several bacteria from the *Enterobacteriaceae* family (6, 10).

β-lactam resistance mediated by inducible chromosomal β-lactamases is closely associated with perturbations in the pathways of peptidoglycan synthesis and recycling that culminate in a high expression of β-lactamase genes (10). In the case of transient induction, the presence of β-lactams increases the products of the peptidoglycan metabolism, which are sensed by regulatory systems, such as the transcriptional activator/repressor AmpR and the two-component system BlrAB, culminating in the activation of *ampC*, *cphA*, and other β-lactamase genes (6, 10). Stable overexpression of these β-lactamase genes is frequently associated with mutations in genes of the peptidoglycan-recycling pathway. For instance, mutations in the *ampD* gene, which encodes the 1,6-anhydro-N-acetylmuramyl-peptide amidase AmpD, are commonly found in several bacterial clinical isolates that are resistant to β-lactam antibiotics by hyperproducing AmpC and other chromosomal β-lactamase enzymes (6, 10–13).

*Chromobacterium violaceum*, a Gram-negative β-proteobacterium commonly found in soil and water worldwide, is an environmental pathogen that causes serious infections in humans and other animals, entering the body after contact of skin lesions with contaminated soil or water (14). Although rare, *C. violaceum* infections in humans show a rapid clinical course and have a high mortality rate in immunocompromised individuals, with bacteria causing bacteremia and damage in several organs, including the liver and spleen (14–17). Misdiagnosis and incorrect antibiotic prescription contribute to the unfavorable outcome, given that clinical *C. violaceum* isolates are intrinsically resistant to some antibiotics, including β-lactams (18–21).

Chromosomally encoded β-lactamases have been studied in many environmental opportunistic pathogens (6, 10), but few studies have investigated the molecular mechanisms of antibiotic resistance in *Chromobacterium* species (22, 23). Genome sequence analysis has revealed the presence of two predicted chromosomal β-lactamase genes, *ampC* and *cmcB*, in *C. violaceum* ATCC 12472 (24, 25) and in clinical *C. violaceum* isolates (26, 27), but their contribution to β-lactam resistance in *C. violaceum* remains to be determined. In this work, we investigated the role and regulation of *ampC* and *cmcB* in response to β-lactams. We found that these genes confer resistance to distinct β-lactams, and their hyperproduction arises from mutations in the amidase AmpD1.

## RESULTS

### Two chromosomal β-lactamases of distinct classes confer high resistance to β-lactam antibiotics in *C. violaceum*

The genome of *C. violaceum* ATCC 12472 (GI: 34105712) has two genes, CV_1310 and CV_3150, which were predicted to encode chromosomal β-lactamase enzymes (24, 25). Our *in silico* analysis with the amino acid sequence alignment using BlastP revealed that CV_1310 has 58% identity with AmpC from *Pseudomonas aeruginosa*, and CV_3150 has 63% identity with CphA from *Aeromonas hydrophila*. We opted to refer to CV_1310 as AmpC, a term commonly adopted in many bacteria for class C β-lactamases. For CV_3150, we designated it as CmcB (*Chromobacterium* Metallo-Carbapenemase B).

**Fig 1:**
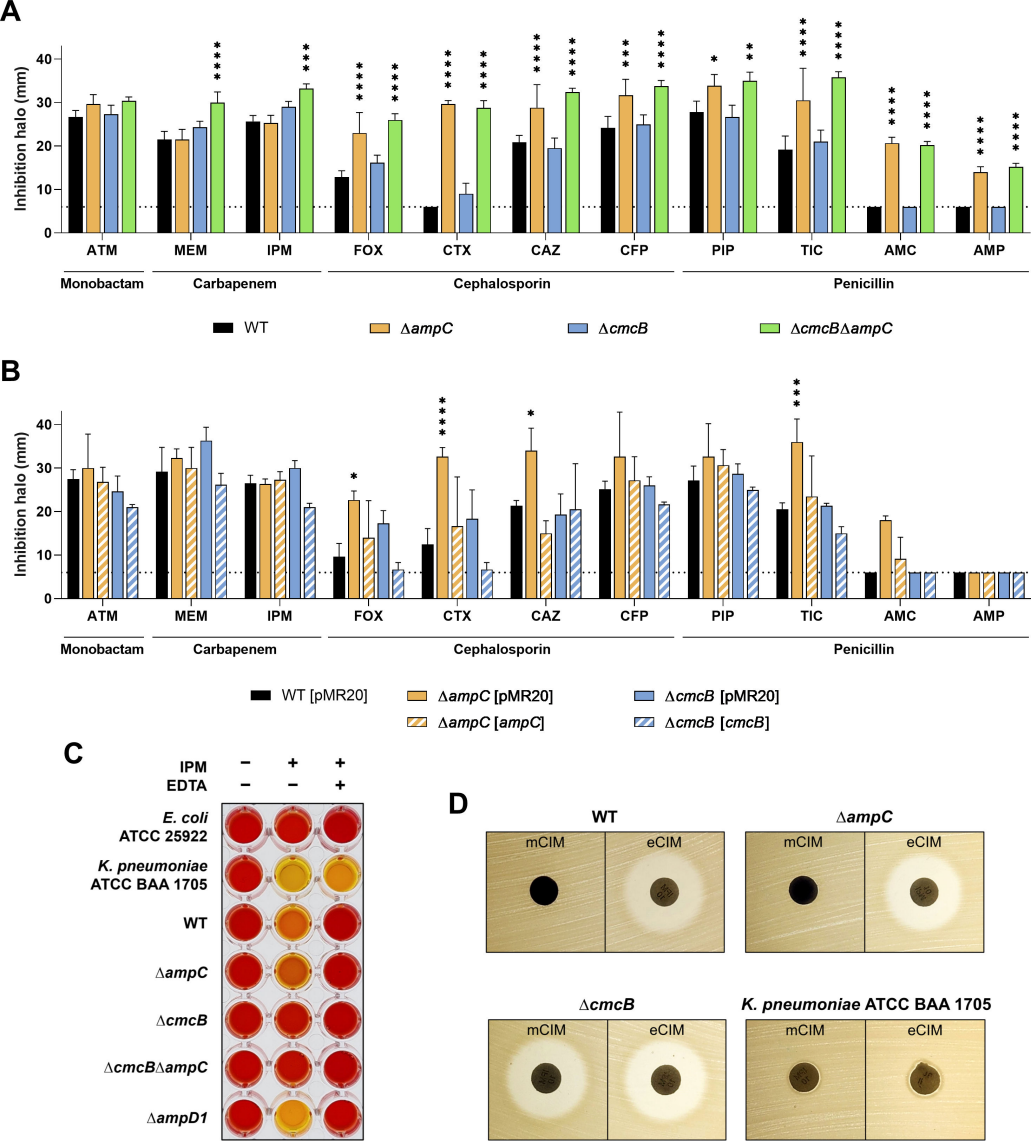
*C. violaceum* harbors two active β-lactamases. (**A and B**) Disk-diffusion assays. (**A**) Deletion of the *ampC* and *cmcB* genes increases *C. violaceum* susceptibility to different β-lactam antibiotics. (**B**) Complementation restores the phenotype of the mutants to a level similar to the wild-type strain. *C. violaceum* WT with and without the empty pMR20 vector were used as controls. Inhibition halos are shown in millimeters. Dotted lines indicate the diameter of the disks (6 mm). ATM, aztreonam; MEM, meropenem; IPM, imipenem; FOX, cefoxitin; CTX, cefotaxime; CAZ, ceftazidime; CFP, cefoperazone; PIP, piperacillin; TIC, ticarcillin; AMC, amoxicillin-clavulanic acid; and AMP, ampicillin. Charts representing the average of the halos measured between the experimental triplicate. ****P < 0.0001; ***P < 0.001; **P < 0.01; *P < 0.05. Two-way analysis of variance (ANOVA) followed by Tukey’s multiple comparisons test. (**C and D**) CarbaNP test, mCIM, and eCIM tests indicate that CmcB is an active metallo-β-lactamase (MBL).

We constructed Δ*ampC*, Δ*cmcB*, and Δ*cmcB*Δ*ampC* null mutant strains and compared their β-lactam resistance profile with that of the wild type (WT). Resistance was evaluated through minimum inhibitory concentration (MIC) assays (Table 1) and disk diffusion tests (Fig. 1A and B), and the mutant phenotypes were further validated by genetic complementation. A Δ*ampC* mutant strain showed increased sensitivity to penicillin, cephalosporin, and monobactam β-lactams but not to carbapenems. On the other hand, a Δ*cmcB* mutant strain was sensitive to the two tested carbapenems (at least in the MIC assays) but not to the other β-lactams. A Δ*cmcB*Δ*ampC* double mutant strain showed the phenotypes observed in each individual mutant strain (Fig. 1A; Table 1). Complementation by providing the *ampC* or *cmcB* genes into each mutant restored or even increased the resistance to the β-lactams, while an empty vector had no effect (Fig. 1B; Table 1). To investigate whether AmpC or CmcB are metallo-carbapenemases, we performed the CarbaNP test (Fig. 1C), the modified carbapenem inactivation method (mCIM), and the EDTA-carbapenem inactivation method (eCIM) using imipenem (Fig. 1D) (28). WT *C. violaceum* showed metal-dependent carbapenemase activity, which was abolished in a Δ*cmcB* but unaffected in a Δ*ampC* strain. As a control, a *Klebsiella pneumoniae* reference strain presented metal-independent carbapenemase activity. Altogether, these data indicate that CmcB is a metallo-β-lactamase conferring resistance to carbapenems, while AmpC is a broad-spectrum β-lactamase that confers resistance to penicillin and cephalosporin β-lactams.

**TABLE 1:** Antibiotic resistance profile of *C. violaceum* β-lactamase mutants^*a*^

Strain	MIC (µg/mL)							
	MEM	IPM	CAZ	CTX	FOX	AMP	AMC	TZP
WT	0.25	2	32	512	64	1,024	1,024	16
WT [pMR20]	0.25	2	32	512	64	1,024	1,024	16
Δ*ampC*	0.25	2	1	2	8	16	16	2
Δ*ampC* [pMR20]	ND	ND	2	4	ND	32	32	4
Δ*ampC* [*ampC*]	0.25	2	128	512	256	1,024	1,024	16
Δ*cmcB*	0.125	1	32	256	32	512	512	8
Δ*cmcB* [pMR20]	0.125	1	64	256	64	512	512	8
Δ*cmcB* [*cmcB*]	4	8	128	512	256	1,024	1,024	16
Δ*cmcB*Δ*ampC*	0.125	1	1	2	8	16	16	1

^*a*^ ND, not determined; WT, wild type; MEM, meropenem; IPM, imipenem; CAZ, ceftazidime; CTX, cefotaxime; FOX, cefoxitin; AMP, ampicillin; AMC, amoxicillin-clavulanic acid; TZP, piperacillin-tazobactam.

### Both *C. violaceum* β-lactamases are induced by β-lactam antibiotics

**Fig 2:**
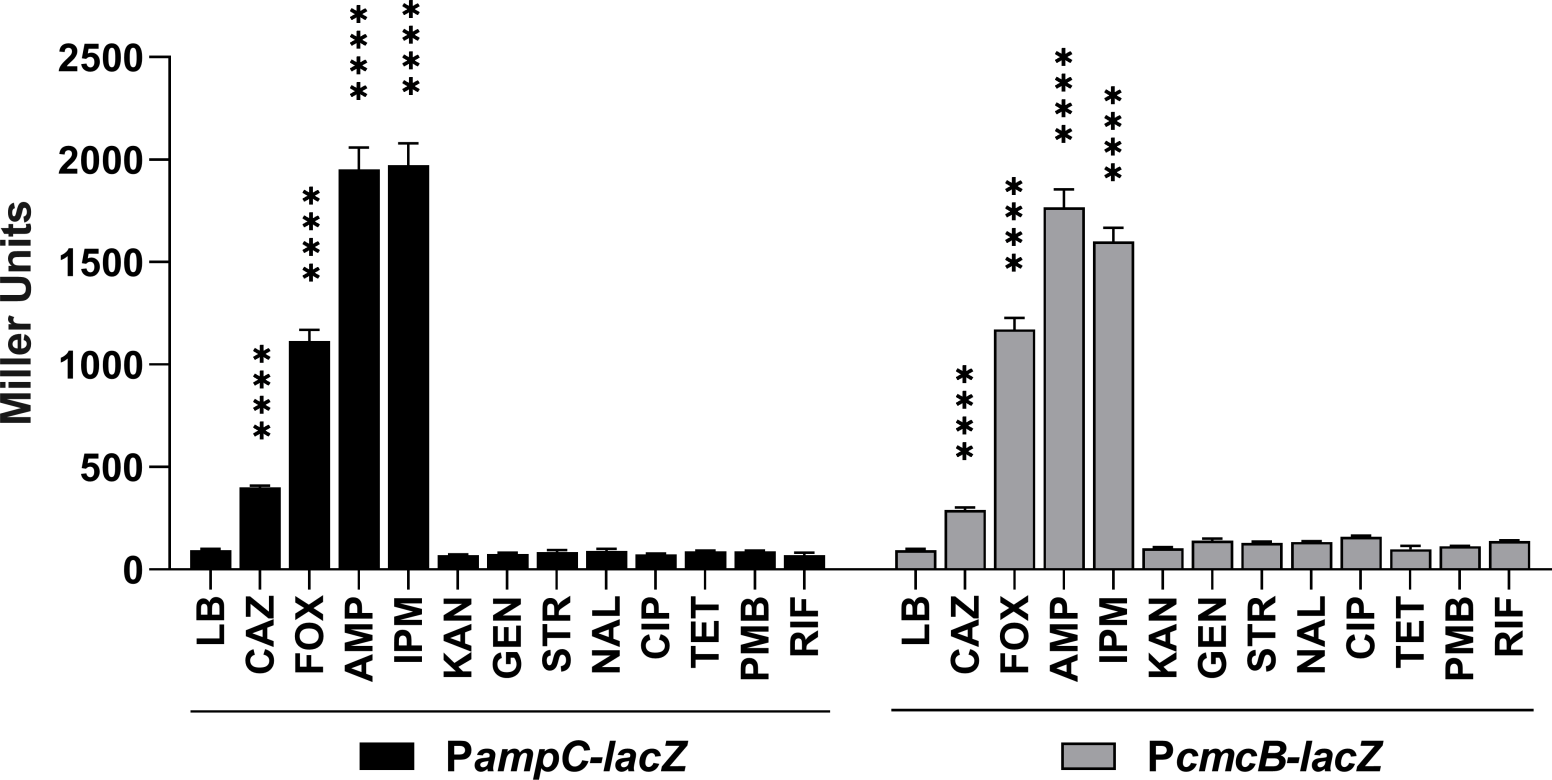
β-lactam antibiotics induce *ampC* and *cmcB* β-lactamases. The expression was measured by β-galactosidase activity assay. *C. violaceum* WT harboring the indicated *lacZ* fusions was cultured in Luria-Bertani (LB) or LB plus the following antibiotics: CAZ, ceftazidime; FOX, cefoxitin; AMP, ampicillin; IPM, imipenem; KAN, kanamycin; GEN, gentamicin; STR, streptomycin; NAL, nalidixic acid; CIP, ciprofloxacin; TET, tetracycline; PMB, polymyxin B; and RIF, rifampicin. The error bars represent the standard deviation of the mean of biological quintuplicates. Stars indicate statistical significance compared to the wild-type strain. ****P < 0.0001. Two-way ANOVA followed by Tukey’s multiple comparisons test.

Some chromosomal β-lactamases are induced in the presence of different β-lactam antibiotics (6, 29). To check whether this is the case for *C. violaceum* β-lactamases, we cloned the promoter regions of *ampC* (P*ampC*) and *cmcB* (P*cmcB*) into a replicative *lacZ*-based reporter vector. The resulting reporter constructs were introduced into the *C. violaceum* WT strain. The promoter activities were quantified by β-galactosidase assays from mid-log phase cultures treated with different antibiotics (Fig. 2). The expression of *ampC* and *cmcB* increased in the presence of β-lactam antibiotics, but while ceftazidime (CAZ) acted as a weak inducer (threefold to fourfold increase), imipenem (IPM), ampicillin (AMP), and cefoxitin (FOX) were strong inducers (10- to 20-fold increase) (Fig. 2). In cultures treated with eight non-β-lactam antibiotics of distinct classes, the promoters of *ampC* and *cmcB* showed a basal expression, comparable with that of untreated cultures (Fig. 2). These results demonstrate that in *C. violaceum*, the expression of both β-lactamases increases when exposed to β-lactam antibiotics.

### Spontaneous mutants isolated on ceftazidime-containing plates are resistant to various β-lactams

**Fig 3:**
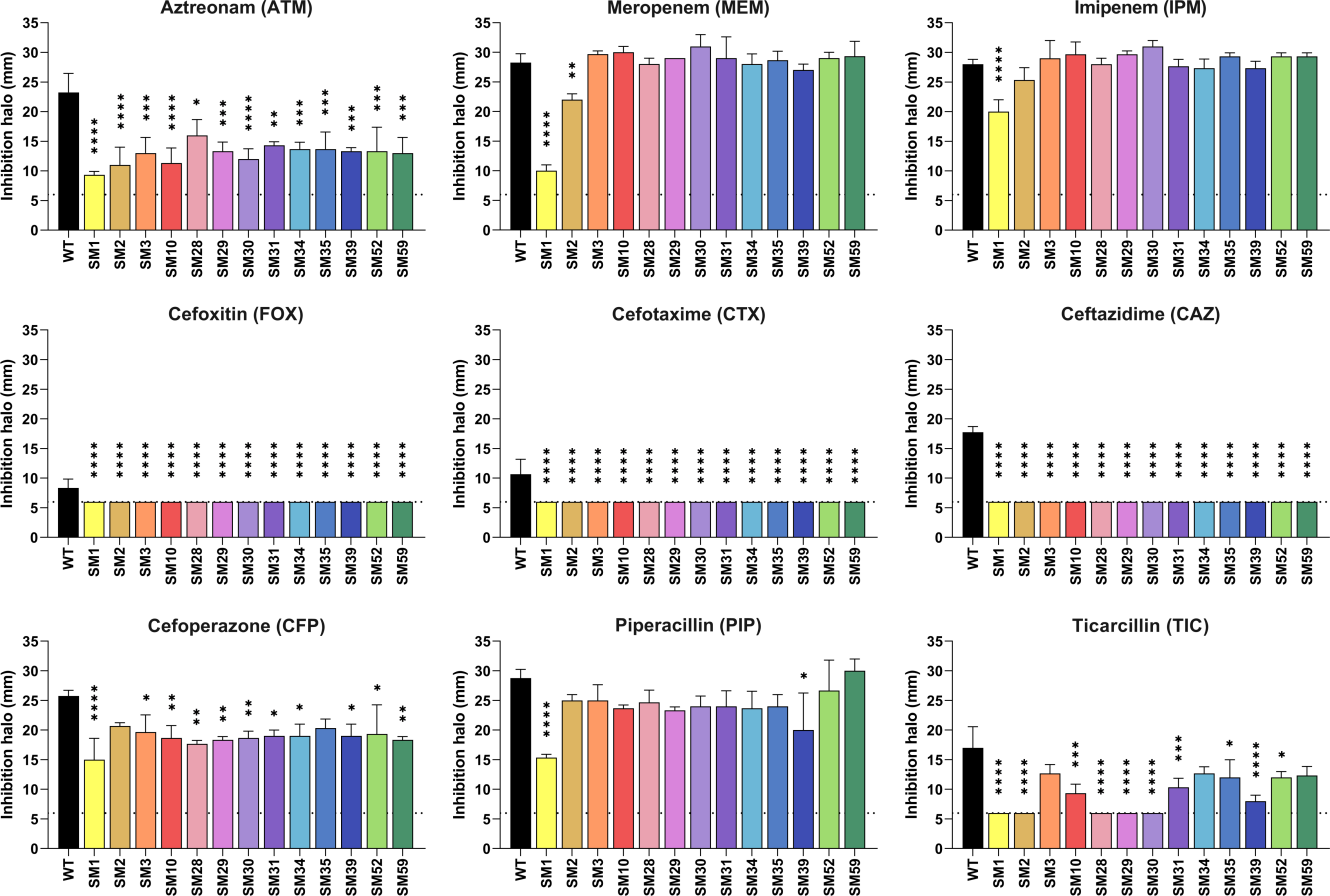
*C. violaceum* spontaneous mutants isolated in ceftazidime are resistant to various β-lactams. Resistance profile of the SM isolates against different β-lactam antibiotics by disk-diffusion assay. Average measurements of the halos from triplicate samples are shown. Halo inhibition is shown in millimeters. Dotted lines indicate the diameter of the disks (6 mm). Stars indicate statistical significance compared to the wild-type strain. ****P < 0.0001; ***P < 0.001; **P < 0.01; *P < 0.05. One-way ANOVA followed by Tukey’s multiple comparisons test.

To identify mutations associated with β-lactam resistance in *C. violaceum*, we isolated spontaneous mutants by plating overnight cultures of the WT strain ATCC 12472 on Mueller-Hinton (MH) agar supplemented with increasing concentrations of CAZ. We chose this cephalosporin because it is a weak inducer of β-lactamases in *C. violaceum* (Fig. 2) and in other bacteria as well (12, 30, 31). A total of 60 colonies were isolated on MH plates containing 80 and 160 µg/mL of CAZ and named SM1 to SM60 (SM stands for spontaneous mutant). After replating the isolates on antibiotic-free MH and determining the MIC by the agar-dilution assay, 13 SM isolates showing MIC values greater than the control were selected (Table 2). We tested the β-lactam resistance profile of the 13 selected SM isolates by disk diffusion using disks of nine β-lactam antibiotics (Fig. 3). The isolates SM1 and SM2 were highly resistant to all tested antibiotics. All the other SM isolates showed increased resistance to most of the tested β-lactams, except for the carbapenems (Fig. 3). These data indicate that the 13 spontaneous mutants isolated in CAZ are resistant to several β-lactam antibiotics. Except for SM1, the SM isolates had a growth profile similar to that of the WT strain (Fig. S1A) and showed little or no change in viability (Fig. S1B).

**TABLE 2:** Ceftazidime MIC of *C. violaceum* WT and SMs

Strain	MIC CAZ (µg/mL)
WT	32
SM1	1,024
SM2	512
SM3	512
SM10	512
SM28	512
SM29	512
SM30	512
SM31	512
SM34	512
SM35	512
SM39	512
SM52	512
SM59	512

### Except for SM1, all spontaneous mutants overexpress both β-lactamases

**Fig 4:**
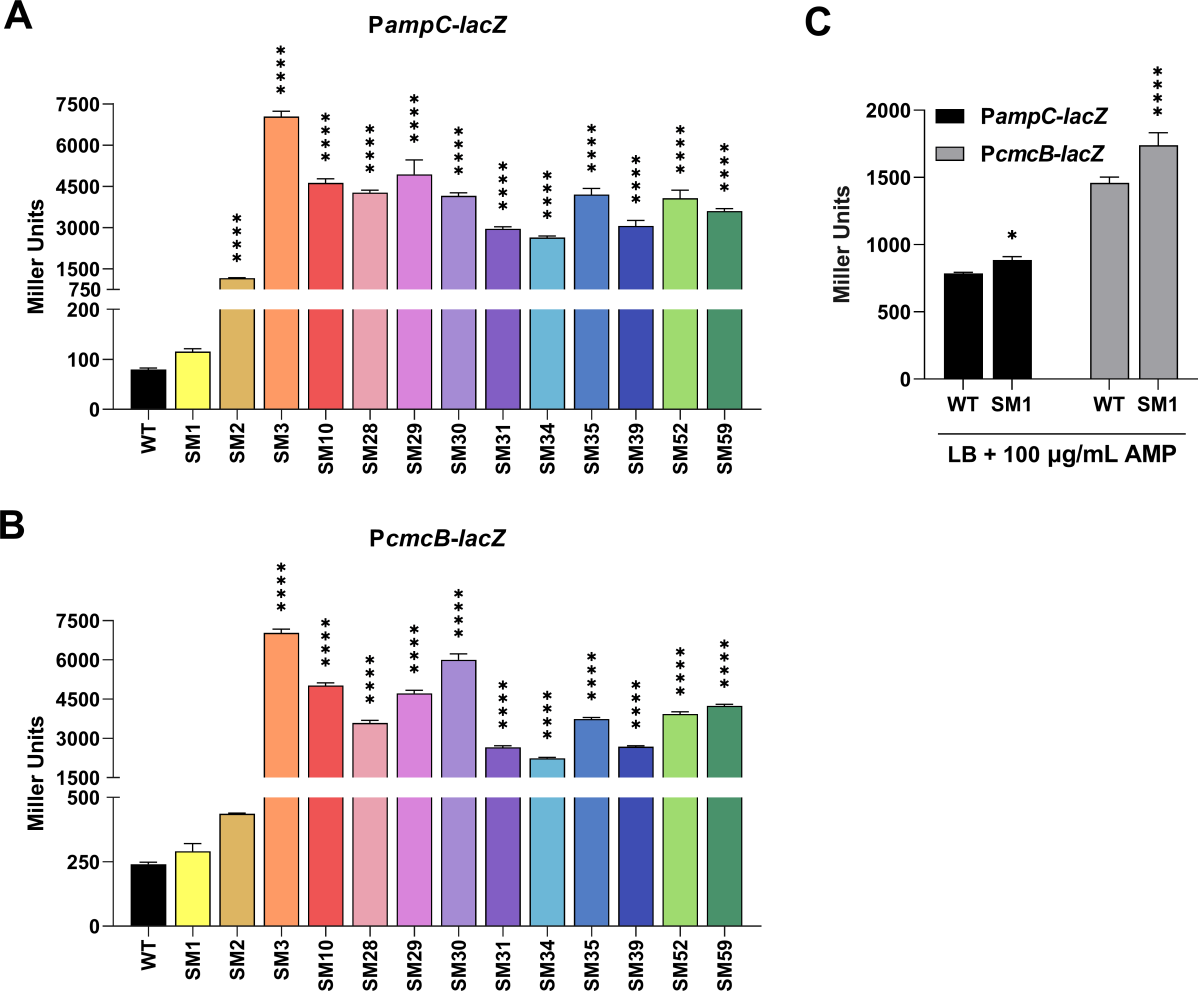
Most spontaneous mutants overexpress both β-lactamases. (**A and B**) Promoter activity of both β-lactamases in LB, and (**C**) LB in the presence of 100 µg/mL AMP. β-galactosidase assays were carried out on WT and SM isolates harboring the P*ampC-lacZ* or P*cmcB-lacZ* fusions. The error bars represent the standard deviation of the mean of a biological quintuplicate. Stars indicate statistical significance compared to the wild-type strain. ****P < 0.0001; *P < 0.05. One-way ANOVA followed by Tukey’s multiple comparisons test.

To associate the β-lactam antibiotic resistance profile of the SM isolates with the expression of the AmpC and CmcB β-lactamases, we carried out β-galactosidase activity assays using the P*ampC* and P*cmcB* transcriptional fusions in the SM isolates (Fig. 4). Except for SM1, the *ampC* and *cmcB* promoters were highly expressed in all SM isolates in comparison with the basal activity found in the WT. The SM2 isolate showed a modest expression compared with the high expression of the other SM isolates (Fig. 4A and B). To check that the *lacZ* fusions were functional in the SM1 isolate, the antibiotic AMP was added to the cultures for 30 minutes. In such conditions, both promoters were highly induced in both the WT and SM1 strains (Fig. 4C). These data indicate that in 12 of the 13 SM isolates with increased resistance to β-lactam antibiotics, overexpression of the *ampC* and *cmcB* β-lactamases occurred. The high resistance of SM1 to β-lactams does not seem to be linked to an altered expression of the AmpC or CmcB β-lactamases.

### Except for SM1 and SM2, all spontaneous mutants have mutations in the *ampD1* gene

**Fig 5:**
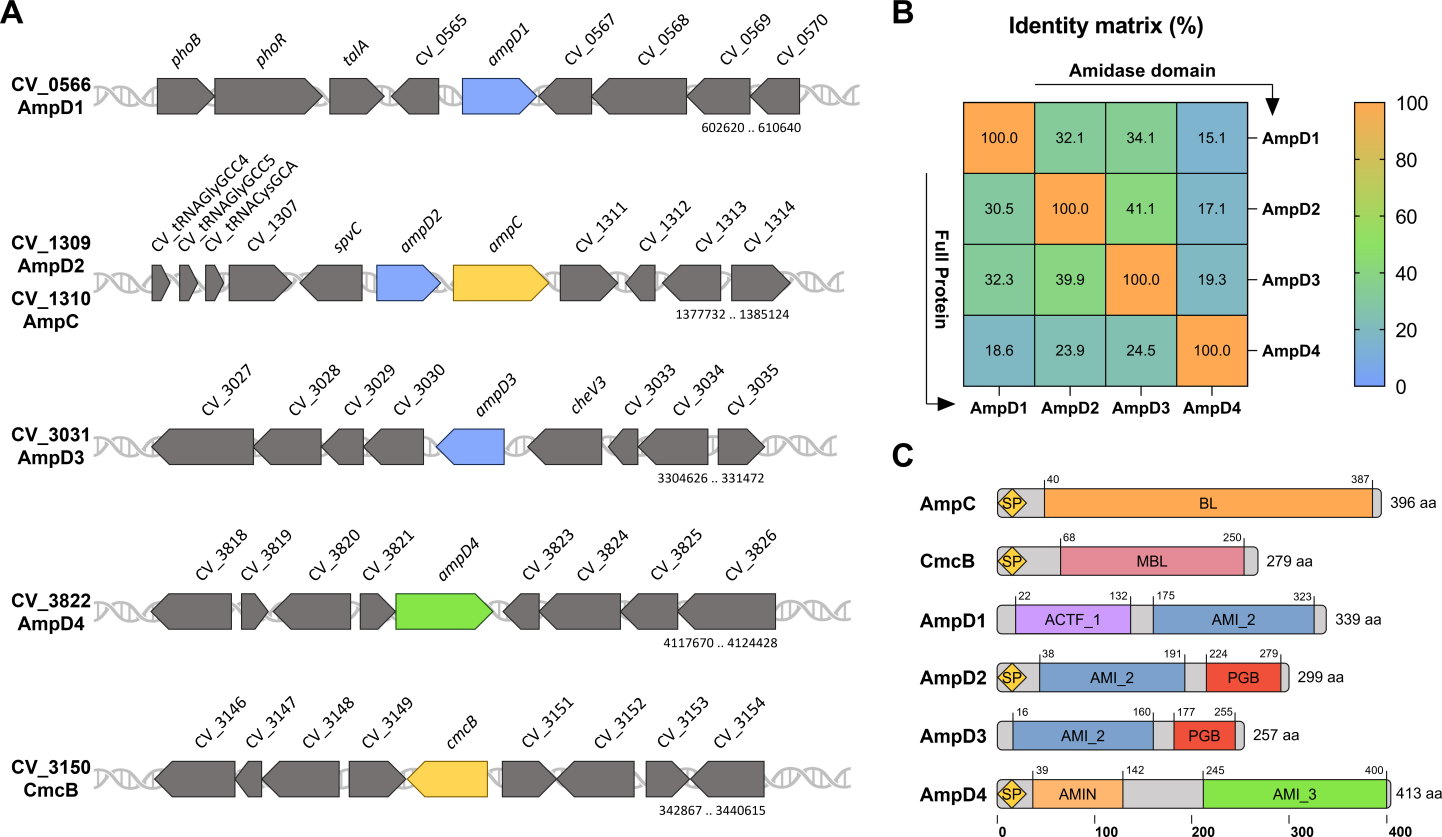
Genomic organization, domain architecture, and multiple alignment of *C. violaceum* amidases. (**A**) Genomic map of the genes encoding β-lactamases and AmpD amidases. (**B**) Similarity matrix between the four *C. violaceum* amidases based on multiple amino acid alignment using Clustal Omega (https://www.ebi.ac.uk/jdispatcher/msa/clustalo). Alignments were performed using the full-length proteins or only their amidase domains, using the default parameters. (**C**) Domain architecture of the amidases. Each color indicates a domain: SP, signal peptide; BL, β-lactamase; MBL, metallo-β-lactamase; ACTF_1, type 1 acetyltransferase; AMI_2, type 2 amidase; PGB, peptidoglycan-binding; AMI_3, type 3 amidase; AMIN, amidase N-terminal domain. Signal peptides (Sec for AmpC and CmcB; Tat for AmpD2 and AmpD4) were predicted using SignalP 6.0 (https://services.healthtech.dtu.dk/services/SignalP-6.0/). The genes have the following entries in Kyoto Encyclopedia of Genes and Genomes, NCBI, and UniProt databases: AmpD1, CV_0566, CV_RS02775, Q7P0K1; AmpD2, CV_1309, CV_RS06380, Q7NYG5; AmpD3, CV_3031, CV_RS23055, Q7NTM3; AmpD4, CV_3822, CV_RS18925, Q7NRF9; AmpC, CV_1310, CV_RS06385, Q7NYG4; CmcB, CV_3150, CV_RS15465, Q7NTA9.

Given the importance of the amidase AmpD in the peptidoglycan recycling process and its close relationship with β-lactam resistance (12, 32, 33), we investigated the occurrence of mutations in *ampD* in the SM isolates. We found three AmpD paralogs in *C. violaceum* that we named as *ampD1*, *ampD2*, and *ampD3*. In the genome, the gene *ampD2* is located next to *ampC* (Fig. 5A). The three AmpD proteins shared 30% to 40% similarity (Fig. 5B) and have an amidase-2 domain (AMI_2; PF01510) (Fig. 5C), found in *Escherichia coli* (AmpD and AmiD) and *P. aeruginosa* (AmpD, AmpDh2, and AmpDh3) (34). Interestingly, in the original annotation, *ampD1* of *C. violaceum* (CV_0566) has a large intergenic region, which when translated in the Expasy translate tool, revealed the existence of a predicted N-terminal N-acetyltransferase domain (Pfam domain ACTF_1) (Fig. 5C; Fig. S2). A potential fourth amidase in *C. violaceum*, CV_3822 (AmpD4), possesses an amidase-3 domain (AMI_3; PF01520) and shows low similarity to the other three AmpDs (18% to 25% in full-length and 15% to 19% in amidase domain alignments) (Fig. 5C). Signal peptide predictions suggest that AmpD1 and AmpD3 are cytoplasmic, a hallmark of AmpDs, while AmpD2 and AmpD4 are exported to the periplasm (Fig. 5C).

**Fig 6:**
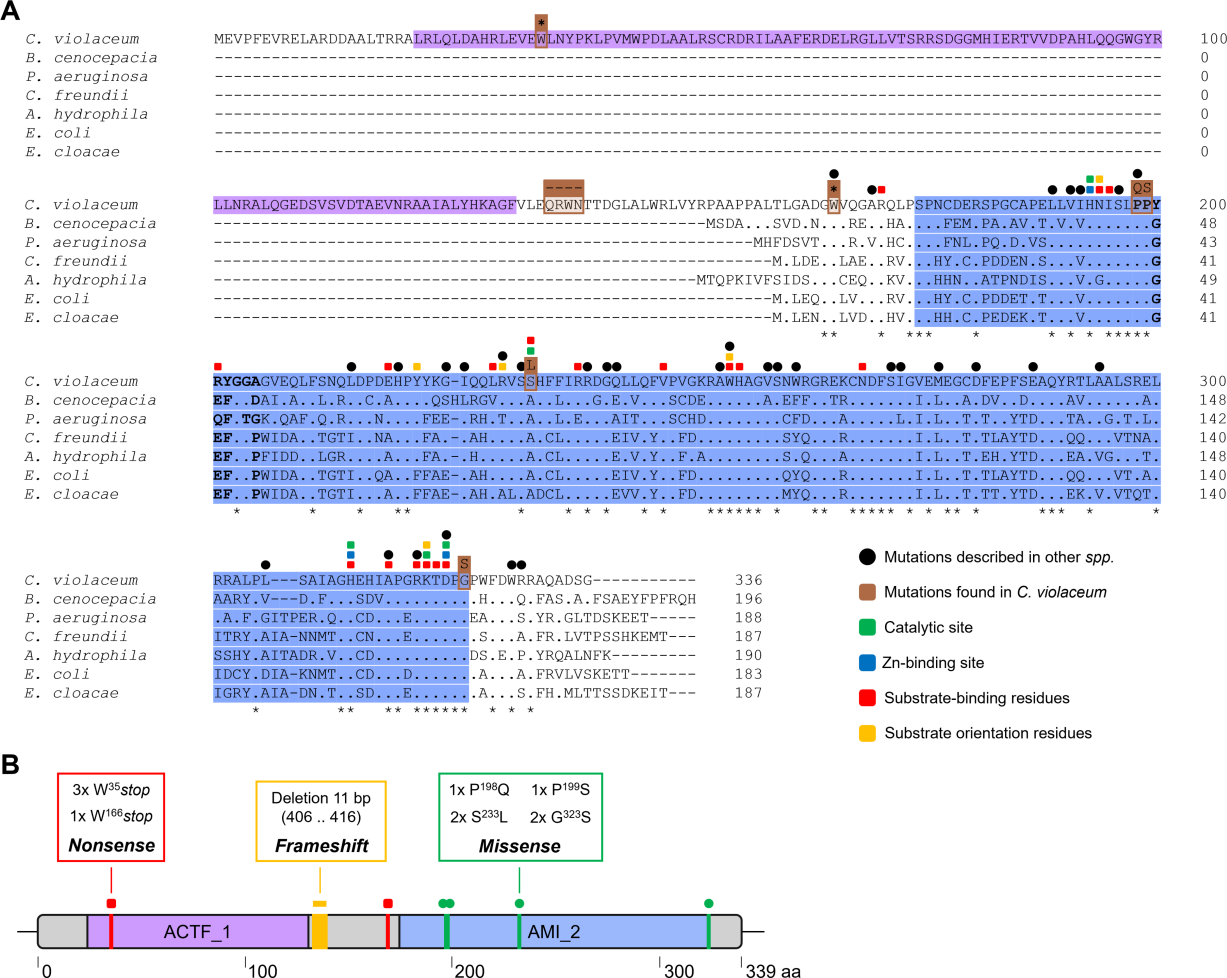
Sequence alignment of AmpD amidases. (**A**) Multiple amino acid alignment performed with Clustal Omega. AmpD1 from *Chromobacterium violaceum* ATCC 12472 (Q7P0K1_CHRVO) was aligned with AmpD sequences from different bacteria, showing the following identity percentages: *Burkholderia cenocepacia* J2315 (B4E5W0_BURCJ, 57%), *Pseudomonas aeruginosa* PAO1 (G3XCW9_PSEAE, 55%), *Citrobacter freundii* OS60 (AMPD_CITFR, 53%), *Aeromonas hydrophila* ATCC 7966 (A0KPV3_AERHH, 52%), *Escherichia coli* K12 (AMPD_ECOLI, 52%), and *Enterobacter cloacae* strain 14 (AMPD_ENTCL, 51%). The protein domains are marked in purple (acetyltransferase) and blue (type 2 amidase). Conserved residues are indicated by colored squares. Black circles indicate mutations reported in other bacteria. The brown boxes indicate mutations found in *C. violaceum ampD1*: replaced amino acids shown at the top; dashes indicate a *frameshift* event, and the star indicates the stop codon. Residues in bold correspond to the loop of the conserved r2 region in the amidase domain responsible for changing the inactive and active state. (**B**) Summary of mutations found in *ampD1* in *C. violaceum* spontaneous CAZ-resistant mutants.

The paralog genes *ampD1*, *ampD2*, and *ampD3* were sequenced by the Sanger method using DNA from the *C. violaceum* ATCC 12472 as a control and from the 13 SMs isolated in CAZ. The DNA sequences were compared by BLAST against the reference genome of the *C. violaceum* ATCC 12472 strain (24). The alignment shows no mutations in the three *ampD* genes in the WT strain, as expected. No mutations were found in the *ampD2* and *ampD3* genes in any of the SM isolates. Except for SM1 and SM2, several mutations were detected in the *ampD1* gene in all SM isolates (Table 3; Fig. 6). Nonsense mutations were detected in the SM3, SM10, SM52, and SM59 isolates that replaced tryptophan codons with a stop codon (Table 3). These mutations occurred in the N-terminal acetyltransferase domain where they generate truncated versions of inactive AmpD1 proteins (Fig. 6). Another six SM isolates showed missense mutations inside the amidase domain, while SM30 had a *frameshift* due to an 11 bp deletion that generates a stop codon in the middle of the *ampD1* gene (Table 3; Fig. 6). Comparing the mutation profile observed in *ampD* mutants from other bacteria with that found in *ampD1* from *C. violaceum*, we discovered many mutations that have already been described and five novel mutations at different regions of the protein (Fig. 6A). Collectively, our data indicate that out of the 13 SMs isolated in CAZ, 12 overexpressed *ampC* and *cmcB,* and 11 had point mutations in the *ampD1* gene (CV_0566). These data suggest that AmpD1 plays a greater role than the other two AmpD paralogs in β-lactam resistance in *C. violaceum*.

**TABLE 3:** Mutations in *ampD1* of CAZ spontaneous mutants^*a*^

Strain	Mutation	Alteration	Position	Classification
Deletion*—ampD1* (CV_0566)				
SM30	Frameshift	−11 pb	(406 .. 416)	
Substitutions*—ampD1* (CV_0566)				
SM10	Nonsense	T**G**G → T**A**G	W35*	Aromatic → Stop codon
SM52	Nonsense	T**G**G → T**A**G	W35*	Aromatic → Stop codon
SM59	Nonsense	T**G**G → T**A**G	W35*	Aromatic → Stop codon
SM3	Nonsense	T**G**G → T**A**G	W166*	Aromatic → Stop codon
SM28	Missense	C**C**G → C**A**G	P198Q	NP/Aliphatic → PNC
SM34	Missense	**C**CT → **T**CT	P199S	NP/Aliphatic → PNC
SM29	Missense	T**C**G → T**T**G	S233L	PNC → NP/Aliphatic
SM35	Missense	T**C**G → T**T**G	S233L	PNC → NP/Aliphatic
SM31	Missense	**G**GT → **A**GT	G323S	NP/Aliphatic → PNC
SM39	Missense	**G**GT → **A**GT	G323S	NP/Aliphatic → PNC

^*a*^ Analysis carried out on the NCBI database with the Blast tool, using the Blastn and Blastx options. In bold are the bases changed by the mutations. SM, spontaneous mutant; *, stop codon; PNC, polar non-charged; NP, nonpolar.

### Mutations in *ampD1*, but not in *ampD2* and *ampD3*, increase resistance to β-lactams via overexpression of β-lactamases in *C. violaceum*

**Fig 7:**
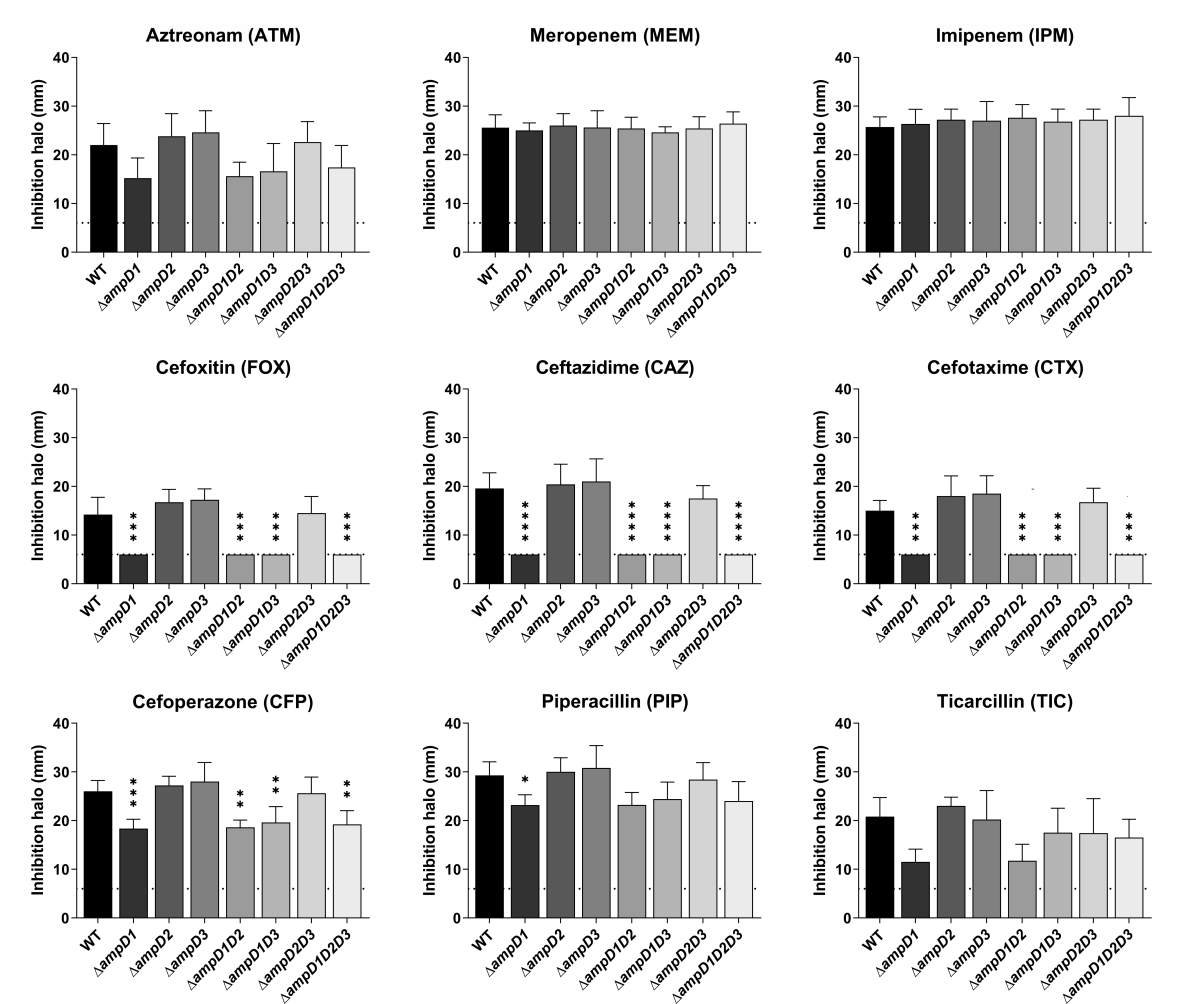
Null mutants with *ampD1* deletion show increased resistance to β-lactams. Resistance profile of the amidase null mutants against different β-lactam antibiotics by disk-diffusion assay. Average measurements of the halos from triplicate samples are shown. Halo inhibition shown in millimeters. Dotted lines indicate the diameter of the disks (6 mm). All the mutant strains were compared with the wild-type strain. ****P < 0.0001; ***P < 0.001; **P < 0.01; *P < 0.05. One-way ANOVA followed by Tukey’s multiple comparisons test.

**Fig 8:**
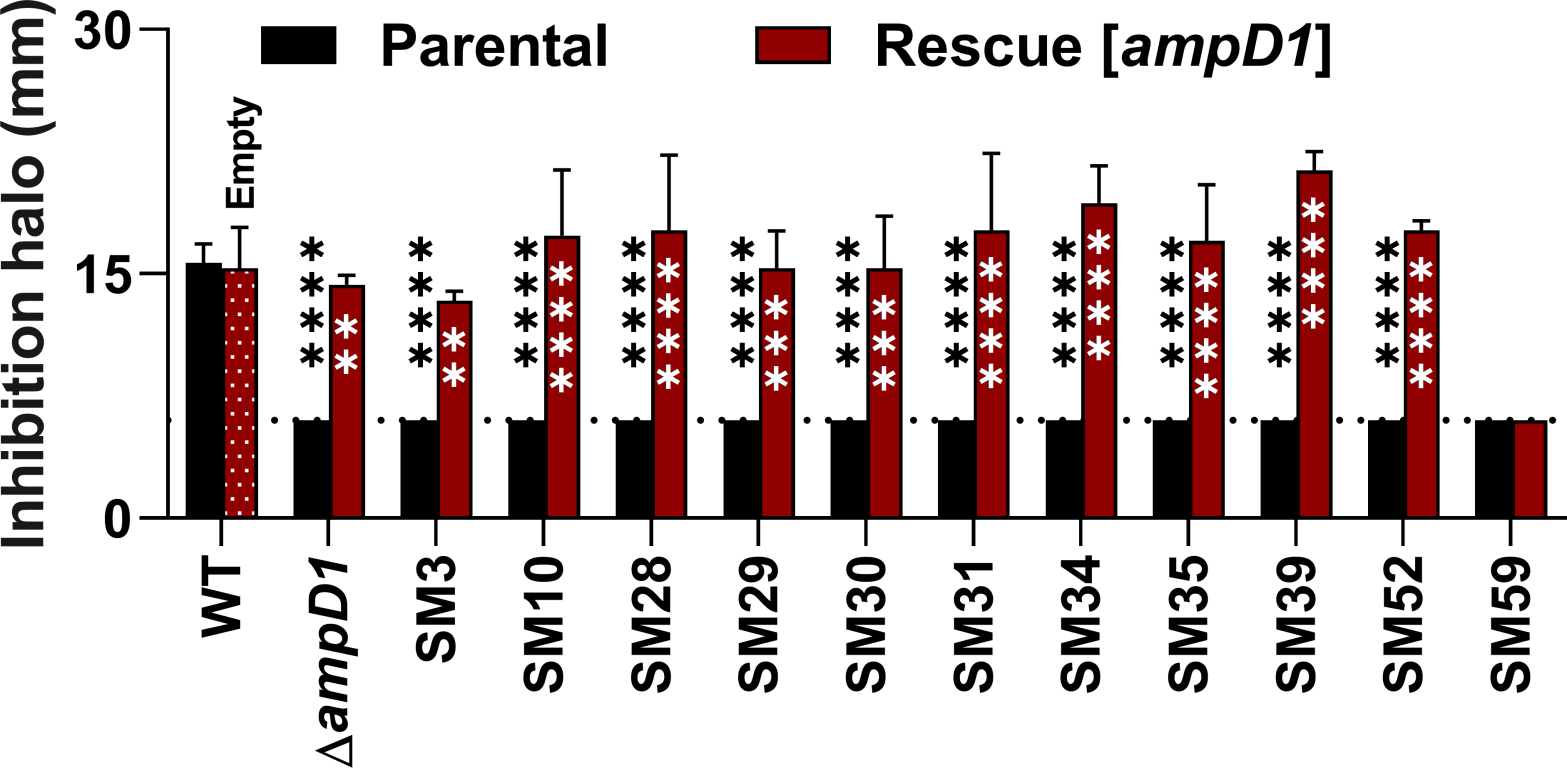
Complementation with the *ampD1* gene rescues the CAZ susceptibility of null and SM strains. The Δ*ampD1* null mutant and all SMs harboring mutation in *ampD1* (parental) were complemented with the *ampD1* gene cloned into the pMR20 vector (rescue). Disk-diffusion assays for CAZ were performed from triplicate samples. *C. violaceum* WT with and without the empty pMR20 vector were used as controls. Inhibition halos presented in millimeters. Dotted lines indicate the diameter of the disks (6 mm). Black asterisks indicate comparisons with the wild-type strain, while white asterisks indicate the comparison in the same strain. ****P < 0.0001; ***P < 0.001; **P < 0.01. Two-way ANOVA followed by Tukey’s multiple comparisons test.

To further investigate the role of the three *C. violaceum* AmpD paralogs in β-lactam resistance, we constructed single null mutants Δ*ampD1*, Δ*ampD2*, and Δ*ampD3*, double mutants Δ*ampD1D2*, Δ*ampD1D3*, and Δ*ampD2D3*, and a triple mutant Δ*ampD1D2D3*. Disk diffusion assays indicated that only mutants with *ampD1* deleted (Δ*ampD1*, Δ*ampD1D2*, Δ*ampD1D3*, and Δ*ampD1D2D3*) had increased resistance to the tested β-lactam antibiotics, if compared with the WT. None of the mutant strains showed a resistance phenotype to the tested carbapenems (IPM and MEM) (Fig. 7). These data are consistent with the β-lactam resistance phenotypes observed for the SM isolates harboring point mutations in *ampD1* (Fig. 3). We also inserted the *ampD1* gene cloned into a vector in Δ*ampD1* and all 11 SM isolates harboring a point mutation in *ampD1*. In all these complemented strains, the increased resistance of the mutants to CAZ was rescued to patterns like those observed for the WT strain, except for SM59 (Fig. 8). These data indicate that the increased resistance to β-lactams in the *ampD1* null mutant and SM isolates (except SM59) is exclusively due to mutations in the *ampD1* gene.

The agar-dilution test to determine the MIC was also carried out for some selected strains (Table 4). As expected from the previous tests, all the mutants with deleted *ampD1* showed resistance to most of the tested antibiotics, if compared with the WT strain, with MIC values at least 5 and 4 times higher for CAZ and FOX, respectively. No resistance phenotype was observed in the mutants of other amidases. A representative spontaneous mutant, the SM3 isolate (nonsense mutation W166stop in *ampD1*), showed MIC values similar to the Δ*ampD1* mutant. In all cases, by providing the *ampD1* gene but not the empty vector, the sensitivity phenotype was rescued (Table 4). We also evaluated the MIC for the other SM isolates complemented with *ampD1* for CAZ, AMP, and IPM. In all cases, the MIC values were the same as those observed in the SM3 complemented strain (data not shown).

**TABLE 4:** Antibiotic resistance profile of *C. violaceum* null *ampD* mutants^*a*^

Strain	MIC (µg/mL)							
	MEM	IPM	CAZ	CTX	FOX	AMP	AMC	TZP
WT	0.25	2	32	512	64	1,024	1,024	16
WT [pMR20]	0.25	2	32	512	64	1,024	1,024	16
Δ*ampD1*	0.5	2	512	1,024	512	1,024	1,024	16
Δ*ampD1* [pMR20]	0.5	2	512	1,024	512	1,024	1,024	16
Δ*ampD1* [*ampD1*]	0.25	2	32	256	64	1,024	1,024	8
Δ*ampD2*	ND	2	32	ND	64	1,024	ND	ND
Δ*ampD3*	ND	2	32	ND	64	1,024	ND	ND
Δ*ampD1D2*	ND	2	512	ND	512	1,024	ND	ND
Δ*ampD1D3*	ND	2	512	ND	512	1,024	ND	ND
Δ*ampD2D3*	ND	2	32	ND	64	1,024	ND	ND
Δ*ampD1D2D3*	ND	2	512	ND	512	1,024	ND	ND
SM3	0.5	2	512	1,024	512	1,024	1,024	16
SM3 [*ampD1*]	0.25	1	128	256	32	512	128	8

^*a*^ ND, not determined; WT, wild type; SM, spontaneous mutant; MEM, meropenem; IPM, imipenem; CAZ, ceftazidime; CTX, cefotaxime; FOX, cefoxitin; AMP, ampicillin; AMC, amoxicillin-clavulanic acid; TZP, piperacillin-tazobactam.

**Fig 9:**
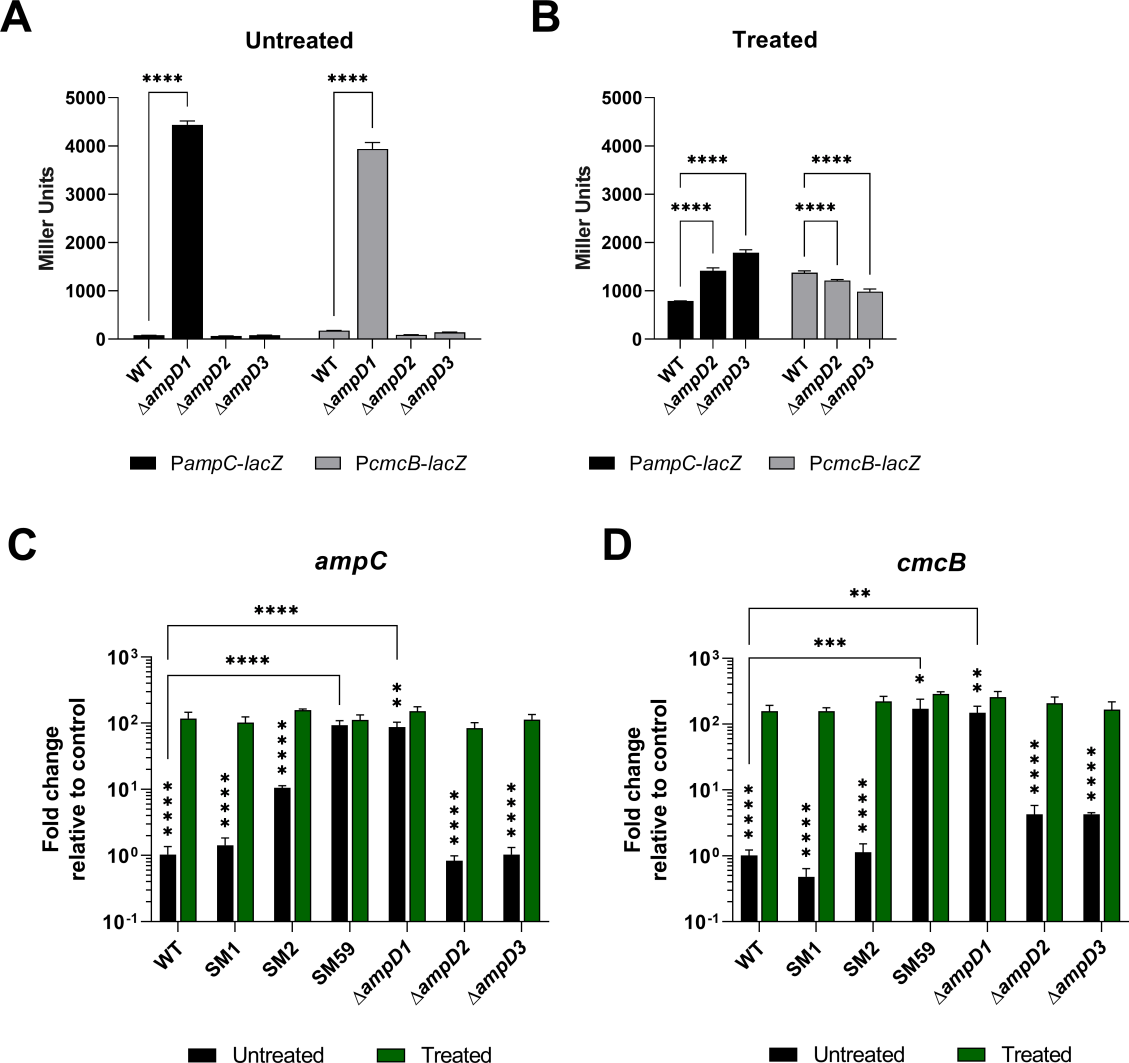
Deletion of *ampD1* increases the expression of β-lactamases in *C. violaceum*. (**A**) The promoter activity of the β-lactamase genes was evaluated in the indicated strains by β-galactosidase activity assay. (**B**) Addition of 100 µg/mL AMP induces promoter expression beyond the basal level. (**C**) Analysis of *ampC* and (**D**) *cmcB* mRNA expression in different strains in control condition (LB) and with 100 µg/mL AMP treatment by RT-qPCR. Assays were performed in biological triplicate. ****P < 0.0001; ***P < 0.001; **P < 0.01; *P < 0.05. Vertical stars indicate statistical significance for the same strain under different conditions. Two-way ANOVA followed by Tukey’s multiple comparisons test was used.

The expression of *ampC* and *cmcB* was evaluated in the three *ampD-*null mutants, Δ*ampD1*, Δ*ampD2*, and Δ*ampD3* (Fig. 9). Both P*ampC* and P*cmcB* showed high β-galactosidase activity in the Δ*ampD1* mutant, indicating overexpression of the two β-lactamases (Fig. 9A), which may explain the β-lactam resistance phenotype in the null mutants and SMs harboring a mutation in the *ampD1* gene. Both promoters had only basal expression in Δ*ampD2* and Δ*ampD3*, comparable to that observed in the WT (Fig. 9A), indicating that AmpD2 and AmpD3 are not involved with the expression of the β-lactamases. The addition of AMP increased *ampC* and *cmcB* expression in all cases, indicating that the reporter fusions in these strains were functional (Fig. 9B). We also investigated the expression of *ampC* and *cmcB* by real-time quantitative PCR (RT-qPCR) (Fig. 9C and D). Consistent with β-galactosidase assays, the SM2, SM59, and Δ*ampD1* mutants showed high expression of *ampC* and *cmcB* in Luria-Bertani (LB) medium with or without AMP, indicating that these strains overexpress the β-lactamases even in the absence of β-lactams. In contrast, the SM1, Δ*ampD2*, and Δ*ampD3* strains showed an inducible expression pattern of *ampC* and *cmcB*, as observed in the WT strain (Fig. 9C and D).

We further investigated the role of the three *C. violaceum* AmpD paralogs and the two β-lactamases in growth, survival, and biofilm formation (Fig. S3). The growth of all mutants was similar to that of the WT strain, except for the triple mutant (Δ*ampD1D2D3*), which showed slower growth (Fig. S3A). In the viability assay, the Δ*ampD1*, Δ*ampD1D2,* and Δ*ampD1D3* mutants showed 1 to 2 log reductions in CFU, if compared with the WT strain (Fig. S3B), with *ampD1* having more dead cells (around 55%, Fig. S3C). Curiously, the Δ*ampD1D2D3* triple mutant had no loss of viability, despite its slower growth (Fig. S3A through C) but their colonies were smaller and displayed a faded purple color. Only the strains harboring the *ampD1* deletion showed a reduction in biofilm formation (Fig. S3D). The β-lactamase mutants had no changes in growth, survival, and biofilm (Fig. S3). Taken together, these data reveal that AmpD1 appears to be the most important amidase for survival and biofilm and that only the deletion of the three amidase genes impacts the growth of *C. violaceum*.

## DISCUSSION

In this work, we demonstrate that *C. violaceum* harbors two active and inducible chromosomally encoded β-lactamases, AmpC and CmcB, which confer resistance to distinct β-lactam antibiotics. Moreover, we provide evidence that mutations in the amidase AmpD1, but not in its paralogs AmpD2 and AmpD3, are responsible for stable overexpression of *ampC* and *cmcB* and increased resistance to β-lactams.

Our results using null *ampC* and *cmcB* mutants in *C. violaceum* (Fig. 1; Table 1) indicate that CmcB is a metallo-β-lactamase conferring resistance to carbapenems, while AmpC is a broad-spectrum β-lactamase that confers resistance to penicillins and cephalosporins. These findings agree with the activity spectrum against β-lactams described for class C and class B2 β-lactamases in other bacteria (6–10). The complementary activities of these two β-lactamases raise a concern because of their widespread co-occurrence in most *Chromobacterium* species (27). On the other hand, a broad-spectrum KPC-like (class A) β-lactamase seems to be restricted to *C. piscinae*, *C. haemolyticum*, and *Chromobacterium* sp. C-61 (35).

We investigated inducers and genetic mutations associated with β-lactam resistance due to changes in β-lactamase expression levels in *C. violaceum*. Our expression data indicate that *ampC* and *cmcB* are induced in response to β-lactams that vary between weak and strong inducers (Fig. 2). These results are consistent with the expression pattern of chromosomally encoded β-lactamases in other bacteria (12, 30, 31). We were able to isolate SMs in the presence of CAZ, a clinically relevant third-generation cephalosporin; 13 of the SMs were further characterized. These SMs showed high MIC values for CAZ (Table 2), increased resistance to most tested β-lactams (Fig. 3), hyperexpression of *ampC* and *cmcB* (12 SMs) (Fig. 4), and they carry mutations of different types in the *ampD1* gene (11 SMs) (Table 3; Fig. 6). These data indicate that mutation in *ampD1* is an important route for the emergence of β-lactam-resistant strains overexpressing AmpC and CmcB β-lactamases in *C. violaceum*. Indeed, mutations in AmpD amidases, which are enzymes involved in peptidoglycan recycling, have been associated with increased expression of β-lactamases and resistance to β-lactam antibiotics in clinical isolates of different bacteria, such as *P. aeruginosa*, *Burkholderia cenocepacia*, *Citrobacter freundii*, *Enterobacter cloacae*, and *E. coli* (11–13, 32, 33, 36–38).

Because we found three AmpD paralogs in *C. violaceum* (Fig. 5), and mutations were detected in *ampD1* but not in *ampD2* or *ampD3* in the SM isolates (Table 3; Fig. 6), we constructed null mutants that lack one, two, or all three *ampD* genes to understand their individual contribution to β-lactam resistance. We provide the following evidence that AmpD1, but not AmpD2 and AmpD3, mediates the regulation and resistance to β-lactams in *C. violaceum*: (i) null mutation in *ampD1*, but not in *ampD2* or *ampD3*, increased the resistance to some β-lactams (Fig. 7; Table 4) and induced high expression of *ampC* and *cmcB* β-lactamases (Fig. 9); (ii) except for SM59, *ampD1* rescued the susceptibility to CAZ when introduced into all the other 10 SM isolates that harbored a point mutation in *ampD1* (Fig. 8). It is curious that the increased resistance in *ampD1* mutants occurred for cephalosporins, some penicillins, and aztreonam, but not for carbapenems, despite (i) these strains had increased expression of *cmcB*, characterized in this work as an active metallo-carbapenemase, and (ii) the high expression of *cmcB* improved resistance to carbapenems in the Δ*cmcB* [*cmcB*] strain (Table 1; Fig. 1). The specific role of AmpD1 in *C. violaceum* resembles that of AmpDs of several bacteria and differs from those that have been described in *P. aeruginosa,* in which sequential inactivation of its three amidases was required to obtain high levels of *ampC* and resistance to β-lactams (39, 40). In this bacterium, the connection between the AmpD mutation and hyperexpression of AmpC is due to a disruption in peptidoglycan recycling products, which act as ligands for the transcription factor AmpR, thereby converting it into an activator of the *ampC* gene (6, 10). Unlike other bacteria, *ampC* and *cmcB* of *C. violaceum* are not adjacent to the genes of the regulators AmpR or BlrA. Our analysis found dozens of candidates with high identity to these regulators in *C. violaceum*.

Remarkably, AmpD1 from *C. violaceum* has an acetyltransferase domain (ACTF_1; PF00583; EC 2.3.1) in its N-terminus belonging to the GNAT (N-acetyltransferases-like-Gcn5) family (Fig. 5). This extra domain is not found in AmpDs characterized in other bacteria (Fig. 6). Amidases with this domain architecture have been annotated and seem to be restricted to bacteria phylogenetically close to *C. violaceum* (data not shown). Further studies are needed to understand the role of the acetyltransferase domain found in the AmpD1 amidase of *C. violaceum*. With respect to other phenotypes of the null *ampD* mutants, the mutant strains with *ampD1* deletion showed a moderate reduction in viability and biofilm formation. Only the triple mutant Δ*ampD1D2D3* showed slower growth (Fig. S3). These minor effects in the absence of the three AmpD amidase paralogs suggest the presence of other amidases in *C. violaceum*. Indeed, a potential fourth amidase in *C. violaceum* (CV_3822) has an amidase-3 domain (PF01520) (Fig. 5) that in *E. coli* is found in the amidases AmiA, AmiB, and AmiC and which are involved in cell division (41, 42). More studies are required to understand the role of the *C. violaceum* amidases in cell division, growth, and survival.

In this work, we demonstrate that the β-lactamases AmpC and CmcB contribute to β-lactam resistance in *C. violaceum*. We identified novel mutations in the unusual amidase AmpD1 that cause stable overexpression of *ampC* and *cmcB*, providing new insights into the molecular mechanisms of β-lactam resistance mediated by chromosomally encoded β-lactamases. Altogether, our data offer an explanation for the limited effectiveness of many β-lactams in treating *C. violaceum* infections. Although this is a limitation in terms of clinical relevance, our findings may pave the way for the development of more effective alternatives. Future studies should search for the transcription factors mediating the regulation of *ampC* and *cmcB* and their relationship with the AmpD1 pathway described in this work. Moreover, genome sequencing of the SM1 and SM2 isolates, which were highly resistant to β-lactams and did not harbor *ampD1* mutations, could reveal alternative mechanisms of β-lactam resistance in *C. violaceum*.

## MATERIALS AND METHODS

### Bacterial strains, plasmids, and growth conditions

The bacterial strains and plasmids used in this work are indicated in Table 5. *E. coli* and *C. violaceum* strains were cultured in LB or MH medium at 37 °C. When required, the media were supplemented with kanamycin (50 µg/mL), tetracycline (10 µg/mL), or ampicillin (100 µg/mL).

**TABLE 5:** Bacterial strains and plasmids used in this work^*a*^

Name	Description	Reference
*Escherichia coli*		
DH5α	Strain used in cloning	(43)
S17-1	Strain used in conjugation for plasmid mobilization	(44)
*Chromobacterium violaceum*		
WT	Wild-type strain (ATCC 12472) with sequenced genome	(24)
WT [pMR20]	Wild-type strain with empty pMR20 vector	This work
Δ*ampC*	Wild-type strain with deletion of the CV_1310 gene (*ampC*)	This work
Δ*ampC* [pMR20]	Δ*ampC* mutant strain with empty pMR20 vector	This work
Δ*ampC* [*ampC*]	Δ*ampC* mutant strain complemented with the *ampC* gene in the pMR20 vector	This work
Δ*cmcB*	Wild-type strain with deletion of the CV_3150 gene (*cmcB*)	This work
Δ*cmcB* [pMR20]	Δ*cmcB* mutant strain with empty pMR20 vector	This work
Δ*cmcB* [*cmcB*]	Δ*cmcB* mutant strain complemented with the *cmcB* gene in the pMR20 vector	This work
Δ*cmcB*Δ*ampC*	Δ*cmcB* mutant strain with combined deletion of the *ampC* gene	This work
Δ*ampD1*	Wild-type strain with deletion of the CV_0566 gene (*ampD1*)	This work
Δ*ampD1* [pMR20]	Δ*ampD1* mutant strain with empty pMR20 vector	This work
Δ*ampD1* [*ampD1]*	Δ*ampD1* mutant strain complemented with the *ampD1* gene in the pMR20 vector	This work
Δ*ampD2*	Wild-type strain with deletion of the CV_1309 gene (*ampD2*)	This work
Δ*ampD3*	Wild-type strain with deletion of the CV_3031 gene (*ampD3*)	This work
Δ*ampD1D2*	Δ*ampD1* mutant strain with combined deletion of the *ampD2* gene	This work
Δ*ampD1D3*	Δ*ampD1* mutant strain with combined deletion of the *ampD3* gene	This work
Δ*ampD2D3*	Δ*ampD2* mutant strain with combined deletion of the *ampD3* gene	This work
Δ*ampD1D2D3*	Δ*ampD1ΔampD2* mutant strain with combined deletion of the *ampD3* gene	This work
Plasmids		
pRK*lacZ*290	Vector for transcriptional fusion to *lacZ*; low copy number; oriV; Tet^r^	(45)
P*ampC*::pRK*lacZ*290	Vector pRK*lacZ*290 with the promoter region of the *ampC* gene	This work
P*cmcB*::pRK*lacZ*290	Vector pRK*lacZ*290 with the promoter region of the *cmcB* gene	This work
pGEM-T-Easy	Cloning vector; Amp^r^	Promega
pNPTS138	Suicide vector; ori ColE1; oriT; ori M13; *nptI*, *sacB*; Kan^r^	M.R.K. Alley
*ampC*::pNPTS138	Vector pNPTS138 with the flanking regions of the *ampC* gene	This work
*cmcB*::pNPTS138	Vector pNPTS138 with the flanking regions of the *cmcB* gene	This work
*ampD1*::pNPTS138	Vector pNPTS138 with the flanking regions of the *ampD1* gene	This work
*ampD2*::pNPTS138	Vector pNPTS138 with the flanking regions of the *ampD2* gene	This work
*ampD3*::pNPTS138	Vector pNPTS138 with the flanking regions of the *ampD3* gene	This work
pMR20	Vector used for complementation of mutants; low copy number and broad host spectrum; RK2 oriV; oriT; Tet^r^	(46)
*ampC*::pMR20	Vector pMR20 with the promoter and coding region of the *ampC* gene	This work
*cmcB*::pMR20	Vector pMR20 with the promoter and coding region of the *cmcB* gene	This work
*ampD1*::pMR20	Vector pMR20 with the promoter and coding region of the *ampD1* gene	This work

^*a*^ Kan, kanamycin; Tet, tetracycline; Amp, ampicillin; r, resistance.

### Construction of *C. violaceum* mutant and complemented strains

In-frame null mutant strains were generated by allelic exchange mutagenesis, as previously described (22, 47). The flanking regions of the gene to be deleted were amplified by PCR using specific primers (Table S1) and cloned into the suicide vector pNPTS138. The transconjugants were plated on LB 16% sucrose, and the null mutants were confirmed by PCR. For genetic complementation, the genes were amplified by PCR using specific primers (Table S1), and cloned into the low copy number vector pMR20 (22, 47). All resulting constructs (for deletion or complementation) were transferred into the *C. violaceum* target strains by conjugation.

### Isolation of ceftazidime-resistant mutants

Spontaneous mutants resistant to ceftazidime were isolated as previously described (12). Briefly, an inoculum of *C. violaceum* ATCC 12472 WT strain from a single colony was incubated overnight in MH broth at 37 °C under agitation. A total of 100 µL of the inoculum was spread on MH agar plates containing increasing concentrations of ceftazidime (40, 80, 160, and 320 µg/mL), and the plates were incubated for 24 hours at 37 °C. Visible and isolated colonies obtained from the 80 and 160 µg/mL MH plates were picked twice in antibiotic-free MH plates and stored in 20% glycerol at –80 °C.

### Antibiotic susceptibility tests

The MIC values were measured by using the agar dilution method according to the recommendations of the Clinical and Laboratory Standards Institute (28). Briefly, fresh colonies grown on MH agar were suspended to OD_600 nm_ 0.1 and diluted to OD_600 nm_ 0.01. Drops of 2 µL of this bacterial inoculum (~10^4^ CFU) were plated on MH agar prepared with or without different serial concentrations of the antibiotics. Bacterial growth was evaluated after incubation at 37 °C for 20 hours.

The resistance profile for several β-lactam antibiotics was evaluated by disk diffusion assays performed as described by CLSI (28). Briefly, fresh colonies grown on MH agar were suspended in 1× phosphate buffered saline (PBS) to OD_600 nm_ 0.1. The bacterial suspension was seeded using a sterile swab on MH plates, and disks with β-lactam antibiotics (Table S2) (BD BBL Sensi-Disc or Thermo Scientific Oxoid) were applied on the top. After incubation at 37 °C for 20 hours, the inhibition halos were measured. In the MIC and disk diffusion assays, the *E. coli* ATCC 25922 strain was used as a control.

To detect metallo-β-lactamase (MBL) activity in *C. violaceum*, we performed mCIM, eCIM, and the CarbaNP test (28). Briefly, the bacterial strains were grown in trypticase soy broth without (mCIM) or with 5 mM EDTA (eCIM). A disk with 10 µg IPM was added, and the cultures were incubated under agitation at 37 °C for 4 hours. The IPM disks were removed from the cultures and applied on the top of an *E. coli* ATCC 25922 indicator strain seeded on MH agar. The plates were incubated for 18–24 hours at 37 °C. The *K. pneumoniae* ATCC BAA 1705 strain was used as a control. For the CarbaNP test, the bacterial strains were lysed using the Express *E. coli* Lysis Reagent kit (New England Biolabs) plus 1 mM PMSF. The supernatants were used in the reaction with the CarbaNP solution (0.05% phenol red, 0.1 mM ZnSO_4_, pH 7.8) with and without the addition of 6 mg/mL imipenem and 10 mM EDTA. The plates were incubated for 2 hours at 37 °C.

### DNA sequencing

The entire *ampD* genes (CV_0566/*ampD1*—1,259 pb; CV_1309/*ampD2*—1,225 pb; and CV_3031/*ampD3*—1,195 pb) were amplified by colony PCR from *C. violaceum* WT and SM isolates and sequenced in both strands. The purified PCR products were quantified in a NanoDrop spectrophotometer (Thermo Scientific). Sanger DNA sequencing reactions were prepared using the BigDye Terminator V3.1 kit (Applied Biosystems), PCR products (40 ng), and suitable primers (Table S1), according to the manufacturer protocol. DNA sequencing was carried out on an ABI 3500XL (Applied Biosystems).

### Transcriptional *lacZ* fusions and β-galactosidase assays

The upstream regions of the *ampC* and *cmcB* genes were amplified by PCR (Table S1) and cloned into the pGEM-T easy plasmid (Promega). The inserts were subcloned into the pRK*lacZ*290 vector to obtain transcriptional fusions of the *lacZ* gene. *C. violaceum* strains containing the reporter plasmids were grown to OD_600 nm_ 1 in LB. The cultures were divided and untreated or treated with the following antibiotics: ceftazidime (CAZ) 30 µg/mL, cefoxitin (FOX) 10 µg/mL, ampicillin (AMP) 100 µg/mL, imipenem (IPM) 1 µg/mL, kanamycin (KAN) 50 µg/mL, gentamicin (GEN) 40 µg/mL, streptomycin (STR) 20 µg/mL, nalidixic acid (NAL) 20 µg/mL, ciprofloxacin (CIP) 1 µg/mL, tetracycline (TET) 3 µg/mL, polymyxin B (PMB) 10 µg/mL, and rifampin (RIF) 10 µg/mL. After 30 minutes, aliquots of the cultures (100 µL) were assayed for β-galactosidase activity using a previously described protocol (47).

### RNA extraction and expression analysis by RT-qPCR

*C. violaceum* strains were cultured in LB at 37 °C under agitation until OD_600 nm_ 1. The cultures were divided and untreated or treated with 100 µg/mL ampicillin (AMP) for 30 minutes. Total RNA was extracted in TRIzol reagent (Invitrogen) and purified using the Direct-zol RNA miniprep plus kit (Zymo), according to the manufacturer’s instructions. The quantity and quality of the RNA was assessed on a denaturing agarose gel and on a Nanodrop spectrophotometer (Thermo Scientific). The cDNA was synthesized using the High-Capacity cDNA Reverse Transcription kit (Applied Biosystems). The quantitative PCR (qPCR) reactions were performed with the PowerUp SYBR Green Master Mix kit (Applied Biosystems), 10 ng of cDNA and 0.5 µM of specific primers (Table S1), using the QuantStudio 3 thermal cycler (Applied Biosystems). The results were analyzed using the QuantStudio Design & Analysis v1.5.2 software with the 2^−ΔΔC*t*^ method (48). Data were normalized to the endogenous *minD* (CV_3376) gene and a reference condition (wild-type strain *C. violaceum* in LB).

### Growth curves

Bacterial growth in LB was monitored over time using absorbance measurements at 600 nm on the BioTek Epoch 2 (Agilent). An overnight pre-inoculum in LB was adjusted to OD_600 nm_ 0.01 in LB, and 150 µL of this dilution was added to a 96-well flat-bottomed microtiter plate. Plates were incubated under 425 CPM orbital shaking (3 mm diameter) at 37 °C for 18 hours. Measurements were taken every hour. These assays were carried out in biological triplicate.

### Survival assays

To calculate colony-forming units per milliliter (CFU/mL), *C. violaceum* cultures were diluted to OD_600 nm_ 0.01 in 5 mL of LB, and grown in a shaker at 37 °C, 250 RPM for 20 hours. After centrifugation, the cultures were serially diluted (1:10) in 1× PBS and plated on LB agar. The colonies were counted after incubating for 20 hours at 37 °C. Alternatively, bacterial survival was evaluated using the LIVE/DEAD BacLight Bacterial Viability kit (Thermo Fisher Scientific), according to the manufacturer’s instructions. Cultures were grown for 20 hours in LB, washed three times with 0.85% NaCl (w/vol), and the cells were stained with 2× LIVE/DEAD solution in a 1:1 ratio for 15 minutes. Samples were prepared on an agarose pad and visualized using a Leica TCS SP5 confocal microscope. Images of randomly obtained fields were analyzed using ImageJ software.

### Static biofilm assay

Biofilm formation was quantified using the crystal violet staining method (47). The *C. violaceum* strains were grown from an OD_600 nm_ of 0.01 in LB in glass tubes under incubation at 37 °C for 24 hours without shaking. The culture was discarded, and 0.1% crystal violet was added for 15 minutes. The tubes were washed, dried, and 33% acetic acid was added. After 1 hour at room temperature, the biofilm was quantified by OD_600 nm_ and normalized by the growth of the culture.

## SUPPLEMENTAL MATERIAL

Supplemental material spectrum.00916-25-s0001.pdf Fig. S1 to S3; Tables S1 and S2.

ASM does not own the copyrights to Supplemental Material that may be linked to, or accessed through, an article. The authors have granted ASM a non-exclusive, world-wide license to publish the Supplemental Material files. Please contact the corresponding author directly for reuse.
