# Supplementary material for: Mutations in ampD cause hyperproduction of AmpC and CmcB β-lactamases and high resistance to β-lactam antibiotics in Chromobacterium violaceum
Source: Microbiol Spectr. 2025 Jun 12;13(8):e00916-25. doi: 10.1128/spectrum.00916-25 (PMC12323641; doi:10.1128/spectrum.00916-25)
Supplement: Supplemental material — Fig. S1 to S3; Tables S1 and S2. [file spectrum.00916-25-s0001.pdf]

SUPPLEMENTAL MATERIAL

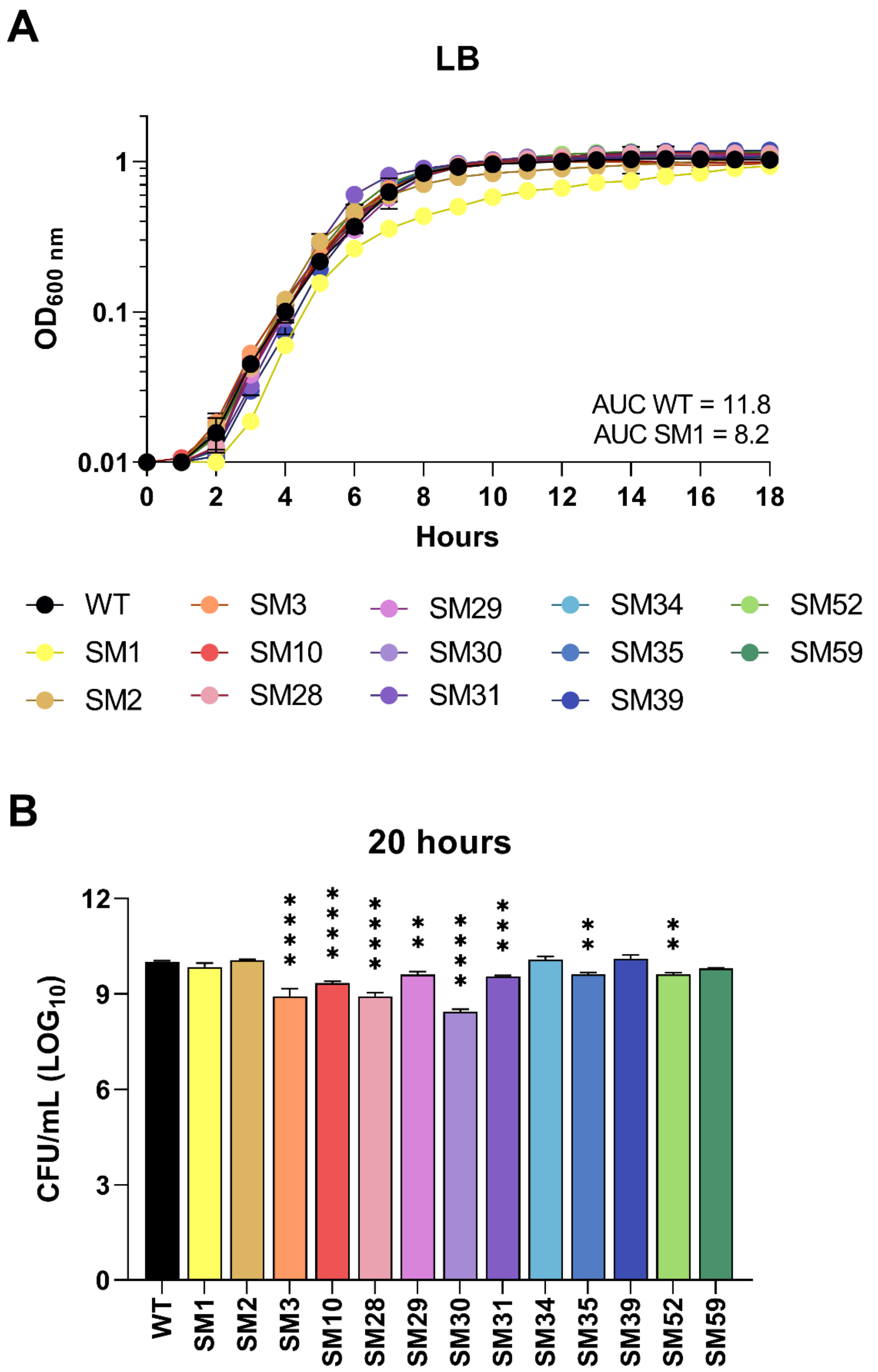

Supplementary Figure 1. Spontaneous mutants have minor change in growth and survival. (A) Growth curves of SMs in LB. The Area Under the Curve (AUC) was evaluated for the bacterial growth curves. **(B)** The viability of the strains was determined by CFU/mL counting. The asterisks indicate statistical significance compared to the wild type strain. Assays were carried out in biological triplicate. ****$p < 0.0001$; ***$p < 0.001$; **$p < 0.01$; * $p < 0.05$. One-way ANOVA followed by Tukey's multiple comparison test was used for the viability test.

```
ATGGAAGTGCCGTTTGAAGTGAGGGAGCTGGCGCGGGACGACGCCGCGCTGACGCGGCGG   60
 M  E  V  P  F  E  V  R  E  L  A  R  D  D  A  A  L  T  R  R
GCCCTGCGGCTGCAGCTGGACGCGCACCGGCTGGAGGTGGAGTGGCTGAATTATCCGAAG   120
 A  L  R  L  Q  L  D  A  H  R  L  E  V  E  W  L  N  Y  P  K
CTGCCGGTGATGTGGCCTGATCTTGCGGCGTTGCGTTCTTGTCGGGACCGGATACTGGCC   180
 L  P  V  M  W  P  D  L  A  A  L  R  S  C  R  D  R  I  L  A
GCCTTCGAAAGGGATGAGCTGCGCGGCCTGCTGGTGACCTCCCGCCGTTCCGACGGCGGC   240
 A  F  E  R  D  E  L  R  G  L  L  V  T  S  R  R  S  D  G  G
ATGCACATCGAACGCACGGTGGTGGATCCGGCGCATCTGCAGCAGGGCTGGGGCTACCGC   300
 M  H  I  E  R  T  V  V  D  P  A  H  L  Q  Q  G  W  G  Y  R
TTGCTGAACCGCGCGCTGCAGGGCGAGGACAGCGTCAGCGTGGACACCGCCGAGGTCAAC   360
 L  L  N  R  A  L  Q  G  E  D  S  V  S  V  D  T  A  E  V  N
CGGGCGGCGATCGCGCTGTACCACAAGGCCGGCTTCGTGCTGGAGCAGCGCTGGAACACC   420
 R  A  A  I  A  L  Y  H  K  A  G  F  V  L  E  Q  R  W  N  T
ACCGACGGCCTGGCCTTGTGGCGGCTGGTTTATCGTCCGGCCGCGCCGCCGGCTTTGACC   480
 T  D  G  L  A  L  W  R  L  V  Y  R  P  A  A  P  P  A  L  T
CTTGGCGCCGACGGCTGGGTGCAGGGCGCGCGGCAGCTGCCGTCGCCCAACTGCGACGAG   540
 L  G  A  D  G  W  V  Q  G  A  R  Q  L  P  S  P  N  C  D  E
CGCAGCCCAGGCTGCGCGCCGGAGCTGCTGGTGATCCATAACATCAGCCTGCCGCCTTAC   600
 R  S  P  G  C  A  P  E  L  L  V  I  H  N  I  S  L  P  P  Y
CGTTACGGCGGCGCGGGCGTAGAGCAGCTGTTTTCCAACCAGCTGGACCCCGATGAGCAC   660
 R  Y  G  G  A  G  V  E  Q  L  F  S  N  Q  L  D  P  D  E  H
CCCTATTACAAGGGGATACAGCAATTGCGGGTGTCGTCGCACTTCTTCATCCGCCGCGAT   720
 P  Y  Y  K  G  I  Q  Q  L  R  V  S  S  H  F  F  I  R  R  D
GGCCAGTTGCTGCAGTTCGTGCCGGTGGGCAAACGCGCCTGGCACGCCGGGGTGTCCAAC   780
 G  Q  L  L  Q  F  V  P  V  G  K  R  A  W  H  A  G  V  S  N
TGGCGGGGGCGCGAGAAGTGCAATGATTTTTCCATCGGCGTGGAGATGGAGGGCTGCGAC   840
 W  R  G  R  E  K  C  N  D  F  S  I  G  V  E  M  E  G  C  D
TTCGAGCCGTTTAGCGAGGCGCAGTACCGGACGCTGGCGGCATTGTCGCGCGAACTGCGC   900
 F  E  P  F  S  E  A  Q  Y  R  T  L  A  A  L  S  R  E  L  R
CGCGCGCTGCCCTTGTCCGCGATCGCCGGCCACGAGCACATCGCGCCGGGGCGCAAGACC   960
 R  A  L  P  L  S  A  I  A  G  H  E  H  I  A  P  G  R  K  T
GATCCCGGTCCCTGGTTCGACTGGCGGCGCGCCCAGGCCGACAGCGGCCTGGGCTTCTGA   1020
 D  P  G  P  W  F  D  W  R  R  A  Q  A  D  S  G  L  G  F  -
```

Supplementary Figure 2. Reannotation of the gene CV_0566 (*ampD1*) in *C. violaceum*. A large intergenic region upstream of CV_0566 was translated in the Expasy translate tool, revealing the existence of a predicted N-terminal N-acetyltransferase domain. The new annotated (ATG) and the old annotated (TTG) start codons are indicated in bold, as well as the stop codon (TGA). The N-acetyltransferase (purple) and the amidase (blue) domains are also highlighted. Numbers refer to the length of the *ampD1* gene in nucleotides.

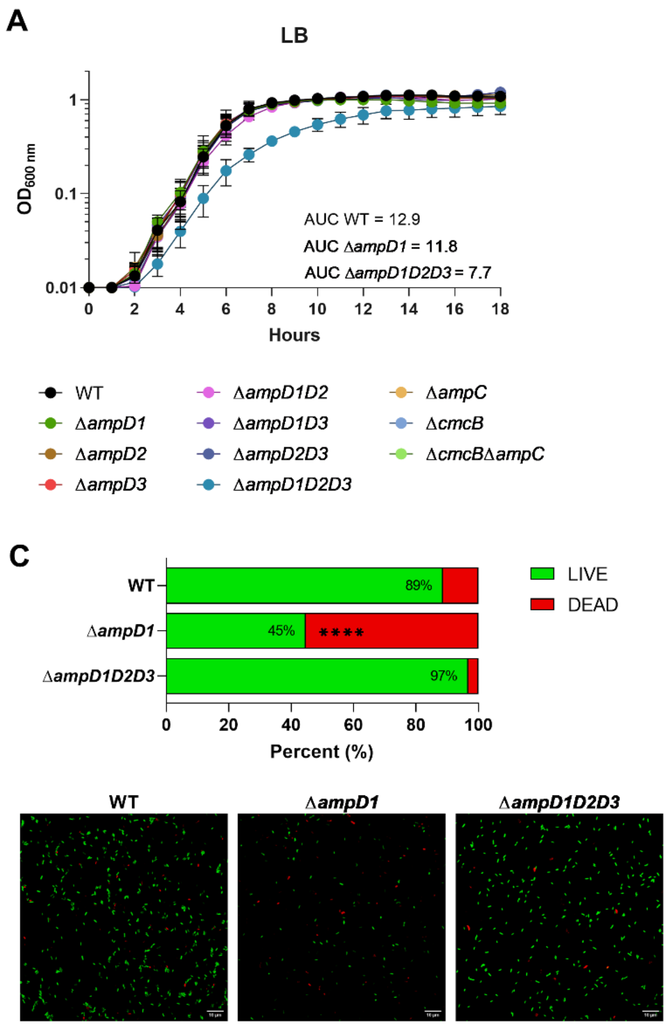

Supplementary Figure 3. Effect of *ampD* deletion on *C. violaceum* traits. (A) Bacterial growth of the wild-type and mutant strains was assessed in LB by measuring absorbance at $OD_{600\ nm}$ for 18 hours. The Area Under the Curve (AUC) was evaluated for the bacterial growth curves. **(B)** The survival of the strains was determined by **(B)** CFU/mL counting and **(C)** LIVE/DEAD assay. **(D)** Biofilm formation was also checked. Stars indicate statistical significance compared to the wild-type strain. Assays were carried out in biological triplicate. ****$p < 0.0001$; ***$p < 0.001$; **$p < 0.01$; * $p < 0.05$. For the analysis of survival was used One-way ANOVA followed by Tukey's multiple comparison test. Two-way ANOVA followed by Tukey's multiple comparison test was used for the LIVE/DEAD assay. Kruskal-Wallis was used for the biofilm test, followed by Dunn's multiple comparison test.

Supplementary Table 1. Primers used in this work.

NAME	SEQUENCE 5' → 3'	APPLICATION
CV0566_Seq_FW	TGGACATGATGCGCCTCAAG	Sequencing of the *ampD1* gene.
CV0566_Seq_RV	CGTTCTACCTGGGCTTCAGC	
CV0566del1*	TACCGGAAGCTTGAACTCCTGCAGCTCGTTGC	Cloning in pNPTS138 with HindIII and BamHI for *ampD1* deletion.
CV0566del2	TACCGGGGATCCCAGCTCCCTCACTTCAAACGG	
CV0566del3	TACCGGGGATCCAAGACCGATCCCGGTCCCTG	Cloning in pNPTS138 with BamHI and EcoRI for *ampD1* deletion.
CV0566del4*	TACCGGGAATTCGTTGGAGCAGAGCGAACTGG	
CV1309_Seq_FW	TGGCTGGGTTTGGCTTTTCC	Sequencing of the *ampD2* gene.
CV1309_Seq_RV	CAGGTCCCGATTGGGAAAGC	
CV1309del1	GATTACAGGATCCGTGGGCGCTCAATTCACCTTG	Cloning in pNPTS138 with BamHI and EcoRI for *ampD2* deletion.
CV1309del2	GATTACAGAATTCGCCGCCAGCGACGATCATTC	
CV1309del3	GATTACAGAATTCGGCGAAGAGGGAGAGCATCC	Cloning in pNPTS138 with EcoRI and SalI for *ampD2* deletion.
CV1309del4	GATTACAGTCGACCCAGTTGCAGCAGCGAGACG	
CV3031_Seq_FW	GCATTGCTCATCACAGACGTG	Sequencing of the *ampD3* gene.
CV3031_Seq_RV	ACACTCCACTTGCGCACTGG	
CV3031del1	TACCGGAAGCTTGCATCAGGATTCCTAACAGGG	Cloning in pNPTS138 with HindIII and BamHI for *ampD2* deletion.
CV3031del2	TACCGGGGATCCAAACCCCTTAATAGCACGATAGC	
CV3031del3	TACCGGGGATCCGCGATCTTGTATGCGCTGGTG	Cloning in pNPTS138 with BamHI and EcoRI for *ampD2* deletion.
CV3031del4	TACCGGGAATTCGCCAGTCCTCCGGGAAACAG	
CV1310del1	GATTACAAGCTTTACAGCCCTGCGATGTCGTC	Cloning in pNPTS138 with HindIII and PstI for *ampC* deletion.
CV1310del2	GATTACCTGCAGGGATTGCATCATCGCGGAAC	
CV1310del3	GATTACCTGCAGATATTGTCGGCGGTGGATCC	Cloning in pNPTS138 with PstI and EcoRI for *ampC* deletion.
CV1310del4	GATTACGAATTCGAATCGAGTTGTAGCCGTAC	

NAME	SEQUENCE 5' → 3'	APPLICATION
CV1310_Comp_FW	GATTACAAGCTTGAAGAGGGAGAGCATCCCTG	Cloning in pMR20 with HindIII and KpnI for *ampC* complementation.
CV1310_Comp_RV	GATTACGGTACCCTCCTGAGCTTGTACTGGCG	
CV1310_RTqPCR_FW	CATTCGACCAGGCGATGGAA	Expression analysis of the *ampC* gene by RT-qPCR, 119 pb.
CV1310_RTqPCR_RV	CTTGCCTGCCTTGGCATAAC	
CV1310_Promot_FW	GATTACGGATCCGAAGAGGGAGAGCATCCCTG	Promoter region of the *ampC* gene used in the pRK*lacZ*290 vector, BamHI and HindIII.
CV1310_Promot_RV	GATTACAAGCTTGGATTGCATCATCGCGGAAC	
CV3150del1	GATTACAAGCTTGTCCCAGGAATAGTTCCAGC	Cloning in pNPTS138 with HindIII and PstI for *cmcB* deletion.
CV3150del2[#]	GATTACCTGCAGTGTGCGCATCTTGATATCCACA	
CV3150del3	GATTACCTGCAGAAAACGCTGGAACGGCTCAAG	Cloning in pNPTS138 with PstI and EcoRI for *cmcB* deletion.
CV3150del4	GATTACGAATTCCATCCTGATCCTGGAGCTGC	
CV3150_Comp_FW[#]	GATTACAAGCTTTCGGTTACGGCAGTTACGGC	Cloning in pMR20 with HindIII and KpnI for *cmcB* complementation.
CV3150_Comp_RV	GATTACGGTACCAGCTTCATCGACGCCAGTCC	
CV3150_RTqPCR_FW	GGAACGCATACTGGAAGTCCA	Expression analysis of the *cmcB* gene by RT-qPCR, 123 pb.
CV3150_RTqPCR_RV	CCGGATAATCGGGAAAGCCG	
M13_FW	GTAAAACGACGGCCAGT	Sequencing of constructions on suicide vector pNPTS138.
M13_RV	AGCGGATAACAATTTCAC	
*lacZ*290UP	TGACGGCTATCACCATCA	Verification of cloning in pRK*lacZ*290 with the M13_FW primer.

Underlined sequences indicate restriction enzyme sites. [*] These primers were also used for cloning in the pMR20 vector. [#] These primers were also used for cloning in the pRK*lacZ*290 vector.

Supplementary Table 2. Antibiotic disks used in this study.

NAME	ACRONYM	CLASSES	QUANTITY (µg)
Ampicillin	AMP	Penicillin	10
Amoxicillin-clavulanic acid	AMC	Penicillin	30
Aztreonam	ATM	Monobactam	30
Cefoperazone	CFP	Cephalosporin 3rd gen.	75
Cefotaxime	CTX	Cephalosporin 3rd gen.	30
Ceftazidime	CAZ	Cephalosporin 3rd gen.	30
Cefoxitin	FOX	Cephalosporin 2nd gen.	30
Imipenem	IPM	Carbapenem	10
Meropenem	MEM	Carbapenem	10
Piperacillin	PIP	Penicillin	100
Ticarcillin	TIC	Penicillin	75
